# Magnetic nanomagnetic nanoparticles combining with Slit2 gene and bone marrow mononuclear cells to improve cognitive dysfunction in rats with chronic cerebral ischemia

**DOI:** 10.7150/ijms.97051

**Published:** 2024-08-19

**Authors:** Nan Wang, Muhui Lin, Wanshu Guo, Yunpeng Cao

**Affiliations:** 1 Department of Neurology Inspection, The First Affiliated Hospital of China Medical University, No. 155 Nanjing Street, Shenyang, Liaoning Province, 110016, China.; 2 Department of Neurology Inspection, The People's Hospital of Liaoning Province, No. 33 wenyi Road, Shenyang, Liaoning Province, 110016, China.

**Keywords:** chronic cerebral hypoperfusion, superparamagnetic iron oxide nanoparticles, bone marrow mononuclear cells, rat

## Abstract

**Purpose:** Cognitive dysfunction caused by chronic cerebral hypoperfusion (CCH) is the leading cause of vascular dementia. Therefore, it is necessary to explore the mechanism that causes cerebral injury and find an effective therapy.

**Methods:** Bone marrow mononuclear cells (BMMNCs) were extracted to detect the activity by CCK-8 kit and verify the transfection efficiency using reverse transcription-quantitative real-time polymerase chain reaction (RT-qPCR). A CCH rat model was established. Superparamagnetic iron oxide nanoparticles (BMPs)-PEI-Slit2/BMMNCs were injected into the tail vein and intervened with an external magnetic field. Hematoxylin and eosin staining was used to observe the pathological changes in brain tissue. The Slit/Robo pathway-related proteins Slit2 and Robo4 were detected by RT-qPCR and Western blotting.

**Results:** The neurological score of the CCH group significantly increased compared with that of the sham group (P<0.05). The levels of brain injury markers S-100β and NSE were significantly higher in the CCH group than in the sham group (P<0.05). Neuronal apoptosis in the frontal cortex and hippocampus of CCH rats significantly increased compared with that of the sham group (P<0.05). The expression levels of Slit2 and Robo4 mRNAs and proteins in brain tissue of CCH rats significantly increased (P<0.05). The neurological function scores of CCH rats treated with BMP-PEI-Slit2/BMMNC significantly increased after Robo4 siRNA administration (P<0.05).

**Conclusion:** BMP combination with the CCH-related gene Slit2 can effectively improve the efficiency of BMMNC transplantation in treatment.

## Introduction

Cognitive dysfunction caused by chronic cerebral hypoperfusion (CCH) is the leading cause of vascular dementia 1. Long-term cerebral hypoperfusion can result in energy dysmetabolism, oxidative stress, the release of inflammatory cytokines, neuronal apoptosis, and cognitive impairment 2,3. CCH-induced vascular dementia can severely impact the patients' quality of life 4. Therefore, it is necessary to explore its mechanism leading to cerebral injury and find an effective treatment.

Bone marrow mononuclear cells (BMMNCs) are mononuclear cell populations in the bone marrow that comprise various stem/progenitor cells, immunoregulatory cells, and other active ingredients, which can promote angiogenesis and improve neurological outcomes by intravenous transplantation 5. However, the technique is hampered by the low percentage of BMMNCs migration into the ischemic brain 6,7. Superparamagnetic iron oxide nanoparticles (BMPs) have strong membrane adsorption capacity and can be adsorbed on the surface of endothelial cells to enter neuronal tissue through the brain capillary wall 8. Astrocytes can swallow iron oxide into cells by endocytosis, allowing BMPs to pass through the blood-brain barrier 9. Polyethylenimine (PEI) has been the most widely studied cationic polymer non-viral gene vector in recent years 10. Combining PEI and BMP with introducing a targeted gene through an external magnetic field can elevate the transfection efficiency and gene expression level 11. Slit is a classical axonal guidance molecule. The Slit-mediated Slit/Robo pathway can regulate endothelial cell migration, pathological angiogenesis, and vascular integrity during ischemic brain injury and promote post-ischemic angiogenesis 12. Slit2 binding to its receptor can attenuate tissue damage and promote recovery after cerebral ischemia 13. Therefore, BMP-PEI-Slit2/BMMNCs may be a potential treatment for CCH-induced brain injury and cognitive impairment, and the underlying mechanisms need to be further investigated.

This study constructed a BMP-PEI-Slit2 complex based on the magnetotactic property, strong DNA adsorption ability of BMPs, high transfection efficiency, and difficult degradation of PEI to investigate whether BMP-modified Slit2 migrated through BMMNCs to induce brain injury and cognitive dysfunction in CCH rats. The study silenced Robo4 to block the Slit/Robo pathway to further investigate whether the ameliorative effect of BMP-PEI-Slit2 BMMNCs on brain injury and cognitive dysfunction in CCH rats was mediated by the Slit/Robo pathway. This study provided a theoretical basis for BMP-PEI-Slit2 BMMNCs as a potential treatment option for cognitive dysfunction caused by CCH.

## Methods

### Preparation of BMP-PEI-Slit2 complex

The prepared and purified BMP and PEI were diluted into 50 μg/mL with normal saline and stored aseptically at 4°C for further use. Then 1 μg of PEI was mixed thoroughly with 1 μg of Slit2, and 0.5 μg of BMP was briefly mixed in an ultrasonic vibrator for 10 to 20 seconds and rapidly placed on ice several times until BMP was fully dispersed and integrated. Afterwards, the premixed PEI-Slit2 solution was added, briefly mixed in an ultrasonic vibrator for 10 to 20 seconds, diluted, mixed in 10-fold phosphate-buffered saline (PBS), and stored at 4°C for preparation.

### BMMNC extraction

All animal experiments have been approved by the animal ethics committee of The First Affiliated Hospital of China Medical University, and great efforts have been made to minize their suffering. BMMNCs were extracted from the femurs and iliac bones of Sprague-Dawley rats by gradient centrifugation. The rats were anesthetized with isoflurane to cut off their iliac bones and hindlimbs. Then the rats were soaked in 75% enthanol folutions five minutes to cut off the hair. Next, the rats were washed with sterile PBS and moved to an ultra-clean bench. The surface muscles of the iliac bone and hind limb were separated to expose the iliac bone, femur, and tibia, place them in PBS, and suck them in the Dulbecco's modified eagle medium (DMEM; Thermo Fisher Scientific, USA) to repeatedly wash the bone marrow into the culture dish until the bone was white and transparent. The same volume of single-cell bone marrow suspension was slowly dripped into Ficoll solution (Sigma-Aldrich, USA) and centrifuged at 3000 rpm for 25 minutes at 4℃. The liquid in the centrifuge tube was divided into three layers, of which the tunica albuginea was BMMNCs. The tunica albuginea was aspirated to add an appropriate amount of DMEM. The cell suspension was repeatedly aspirated, centrifuged at 3000 rpm for five minutes at 4°C, and washed thrice. BMMNCs were diluted to a predetermined concentration. Then 10 μL of the cell suspension was counted on a blood cell counting board and placed on ice for later use.

### CCK-8 assay

Cell viability was determined using CCK-8 assay. The cells were seeded in 96-well plates at 3×103 cells per well. The medium was discarded after an overnight culture to add the CCK-8 detection reagent (Abcam, USA) for an hour of incubation at 37°C. The optical density values at 450 nm were detected with a microplate reader (Thermo Fisher Scientific, USA).

### Reverse transcription-quantitative real-time polymerase chain reaction (RT-qPCR)

Total RNA was extracted from tissues or cells by the Trizol-based method. mRNA was reverse transcribed into cDNA by a reverse transcription kit (Thermo Fisher Scientific, USA). Green®Premix Ex Taq™II (Takara Bio, Japan) was used to detect the expression levels of Slit2 and Robo4 mRNAs. The 2^-∆∆Ct^ calculation was utilized to quantify the relative mRNA expressions using GAPDH as an internal reference.

### Directional induction and differentiation experiments

Directional induction and differentiation experiments were performed when the third passage of BMMNCs grew to 80% of confluence. Serum-free medium with 1 mmol/L of β-sulfuryl ethanol was added and cultured in an incubator. The preinduction solution was removed after 24 hours. The cells were washed three times with PBS and supplemented with a solution containing 20 g/L of DMSO and 5 mmol/L of β-sulfuryl ethanol to continue the induction. The cells were observed microscopically after five hours of induction (Olympus, Japan). MAP2, NeuN, Nestin, NSE, GFAP, and synapsin-1 expressions were examined by immunofluorescence assay below.

### Cellular immunofluorescence assay

Cells were prepared into cell slides, washed with PBS to remove excess culture medium, fixed with 4% paraformaldehyde for 15 minutes, washed with PBS, penetrated with Triton X-100 at room temperature for 15 minutes, blocked with goat serum at room temperature for one hour. Cells were incubated with MAP2, NeuN, nestin, NSE, GFAP, and synapsin-1 antibodies (Abcam, USA) overnight at 4°C, washed three times with PBS, incubated with fluorescent secondary antibodies at room temperature in the dark for one hour, washed three times with PBS, incubated with DAPI staining solution at room temperature in the dark for 30 minutes, washed three times with PBS, mounted with anti-fluorescent quenching blocking reagent, and observed and photographed under a fluorescence microscope (Olympus, Japan).

### Western blotting

Total tissue and cellular proteins were extracted using RIPA lysis buffer (Thermo Fisher Scientific, USA). The protein concentrations were detected using a BCA protein assay kit (Thermo Fisher Scientific, USA). The proteins were separated by sodium dodecyl sulfate-polyacrylamide gel electrophoresis and transferred to polyvinylidene fluoride membranes. Then the membranes were incubated overnight at 4°C using Slit2 and Robo4 primary antibodies (Abcam, USA). Horseradish peroxidase-labeled goat anti-rabbit secondary antibodies (Abcam, USA) were incubated for one hour at room temperature. Color development was performed using an electrochemiluminescence solution and gel imaging system (Bio-Rad, USA). The gray values of the images were calculated using Image J software (USA).

### Grouping and treatment of experimental animals

Fifty Sprague-Dawley rats weighing between 260 and 280g were provided by Liaoning Changsheng Biotechnology Co., Ltd. (China). The project was approved by the Institutional Animal Care and Use Committee of China Medical University. Rats were bred in micro-isolator cages (24℃) under a 12:12 h light-dark cycle with free access to regular chow and water. The rats were randomly divided into sham, CCH, control, BMP-PEI-Slit2/BMMNCs treatment, and Robo4 siRNA (si-Robo4) groups (n=10).

The CCH rat model was established by permanently ligating bilateral common carotid arteries in all groups except for the sham group 14. Rats were anesthetized by intraperitoneal injection of 2% pentobarbital (3 mg/kg) and placed in the supine position. The mouse neck was prepped and disinfected after fixation. A midline cervical incision was made to separate the bilateral common carotid arteries and vagus nerves. The bilateral common carotid arteries were permanently ligated using no. 4 surgical sutures except for the sham group, and the tissues were closed by layer sutures. The BMP-PEI-Slit2/BMMNCs complex that was dissolved in 300 μl of DMEM was transplanted into the tail vein of rats in the BMP-PEI-Slit2/BMMNCs treatment and si-Robo4 groups using a 1 mL syringe. The rat heads were adsorbed with magnets for 30 minutes. Besides, the control group was injected with 300 μl of DMEM. The si-Robo4 group was intracerebrally injected with 10 μl of ROBO4 siRNA (2 μmol/L) before CCH modeling.

### Assessment of neurological function score

Beders' neurological deficit score was used to assess neurological deficits. The scoring criteria were as follows: 0 point represented normal with no symptoms of nerve injury; 1 point represented flexion and inability to straighten fully of the forelimb contralateral to the lesion during tail lifting; 2 points represented circling to the paralyzed side during walking; 3 points meant falling to the contralateral side of the lesion; and 4 points represented loss of consciousness and inability to walk spontaneously.

### Morris water maze

Rats were tested for learning and memory functions by directional navigation and spatial exploration tests in the Morris water maze. The directional navigation experiment lasted five days to calculate the time for rats to find the platform inside the maze. The rats were placed in water facing the pool wall at four entry points, respectively. The time they took to find and climb the platform within three minutes twice daily was recorded as the escape latency, and the walking path during this period was the swimming distance. The time for rats to find and climb the platform within three minutes each time for four consecutive days was the memory performance of rats, and the average performance was calculated. The spatial exploration test was used to detect rats' ability to remember the platform's spatial location after familiarity with it. The platform was removed after the localization navigation test. Then the rat was placed in the pool at any entry point to record the number of times it crossed the original location within 180 seconds.

### Hematoxylin and eosin (HE) staining

The tissue samples fixed in paraformaldehyde were deparaffinized with gradient concentrations of ethanol solutions, cleared by xylene, dipped in wax, and sectioned for HE staining. The samples underwent hematoxylin staining for ten minutes, hydrochloric acid differentiation for 30 seconds, eosin staining for one minute, running water irrigation, dehydration, transparency, and neutral gum mounting to observe histopathological changes in the frontal cortex and hippocampal neuron of the brain under a light microscope.

### Enzyme-linked immunosorbent assay (ELISA)

The S-100β and NSE levels were detected strictly according to the instructions of ELISA kit (Thermo Fisher Scientific, USA). The reaction plate was taken out when the kit was at room temperature. The standard and diluted samples were added to the corresponding reaction plate wells, gently shaken for 30 seconds, and incubated at 37℃ for two hours. Horseradish peroxidase-labeled antibody was added to the plate for incubation at 37°C for one hour. The plate was washed three times, added with biotin-labeled avidin, and incubated at 37℃ for one hour. Then the plate was washed five times, added with TMB substrate, set at 37℃ in the dark for 15 minutes, and added with the stop solution to detect the absorbance at 450 nm by the microplate reader, with the measured absorbance value of the standard as the ordinate and the concentration of the standard as the abscissa. The standard curve was drawn to calculate the sample concentration.

### Terminal dUTP nick-end labeling (TUNEL) staining

TUNEL kit (Thermo Fisher Scientific, USA) was used to detect neuronal apoptosis in the brain. The rat brain tissues were routinely dehydrated, embedded, and sectioned at each time point. The paraffin sections were deparaffinized and hydrated with gradient concentrations of ethanol solutions, soaked in distilled water, dropped with proteinase K, and incubated at room temperature for 30 minutes. After PBS washing, the terminal deoxynucleotidyl transferase and fluorescence labeling solution were added dropwise into the sections and incubated at 37°C for one hour. After PBS washing, the DAPI staining solution was incubated into the sections at room temperature for 30 minutes, mounted with an anti-fluorescent quenched blocking agent, and observed and photographed under a fluorescence microscope.

### Tissue immunofluorescence assay

The brain tissues were fixed in 4% paraformaldehyde, dehydrated in gradient concentrations of ethanol solutions, cleared in xylene, soaked in wax, embedded in paraffin, and sectioned. The paraffin sections were deparaffinized in xylene, hydrated in gradient concentrations of ethanol solutions, washed in distilled water, immersed in PBS, antigen-retrieval with sodium citrate buffer, soaked in PBS, blocked in goat serum at room temperature for one hour, incubated with MAP2 antibody at 4°C overnight, washed three times with PBS, incubated with fluorescent secondary antibody at room temperature in the dark for one hour, washed three times with PBS, incubated with DAPI staining solution at room temperature in the dark for 30 minutes, washed three times with PBS, mounted with anti-fluorescent quenching blocking agent, and observed and photographed under a fluorescence microscope.

### Statistical analysis

SPSS version 22.0 (IBM Inc., USA) was applied for data processing and statistical analysis. The measurement data were expressed as the mean ± standard deviation. The comparisons among multiple groups were performed by one-way analysis of variance. The Tukey's post hoc test was used for pairwise comparisons. A P value of less than 0.05 was considered statistically significant.

## Results

### BMP-PEI-Slit2 induced BMMNC differentiation into neuron-like cells

**Figure 1:**
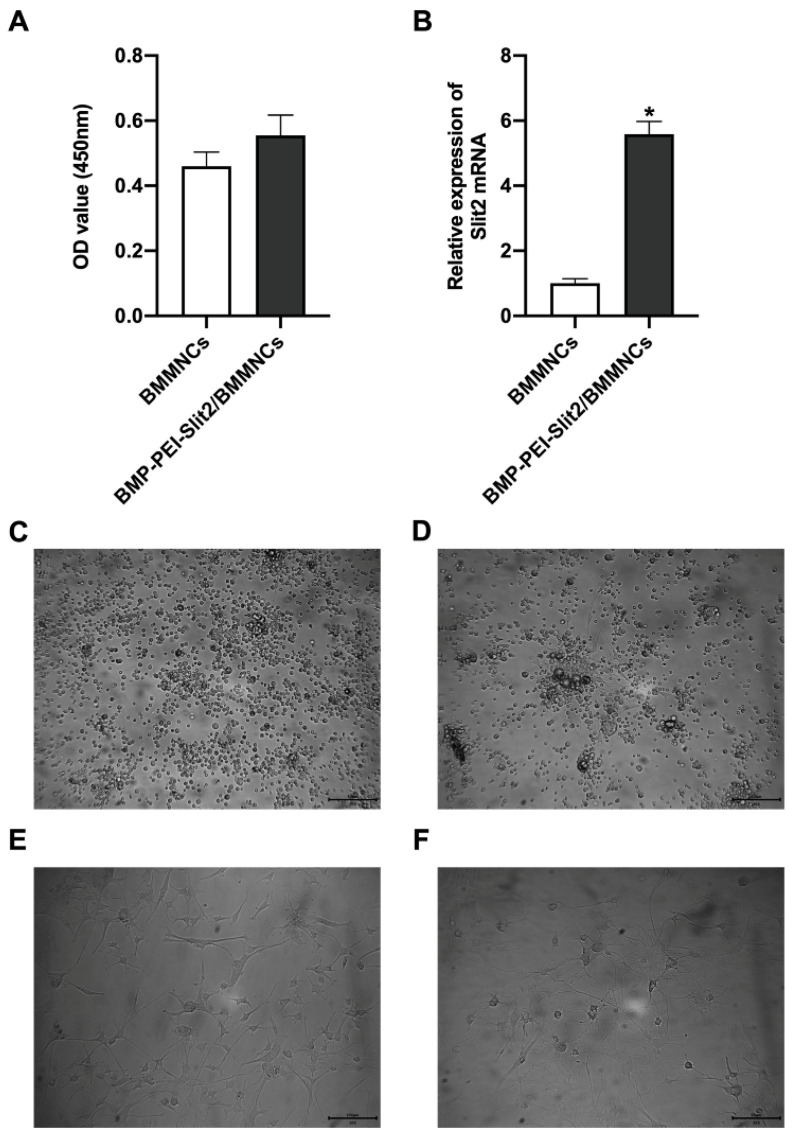
Preparation of BMP-PEI-Slit2 BMMNCs. BMMNC viability after BMP-PEI-Slit2 was co-cultured with BMMNCs for 24 hours detected by CCK-8 (A); and Slit2 mRNA expression detected by RT-qPCR (B). The primary cells (C), the cell after three days of induced culture (D), the cell after seven days of induced culture (E), and the cell after 14 days of induced culture (F) were observed by the inverted microscope. *, *p* < 0.05 compare with BMMNCs group. BMMNC: Bone marrow mononuclear cell; BMP: superparamagnetic iron oxide nanoparticle; PCR: polymerase chain reaction; PEI: Polyethylenimine.

**Figure 2:**
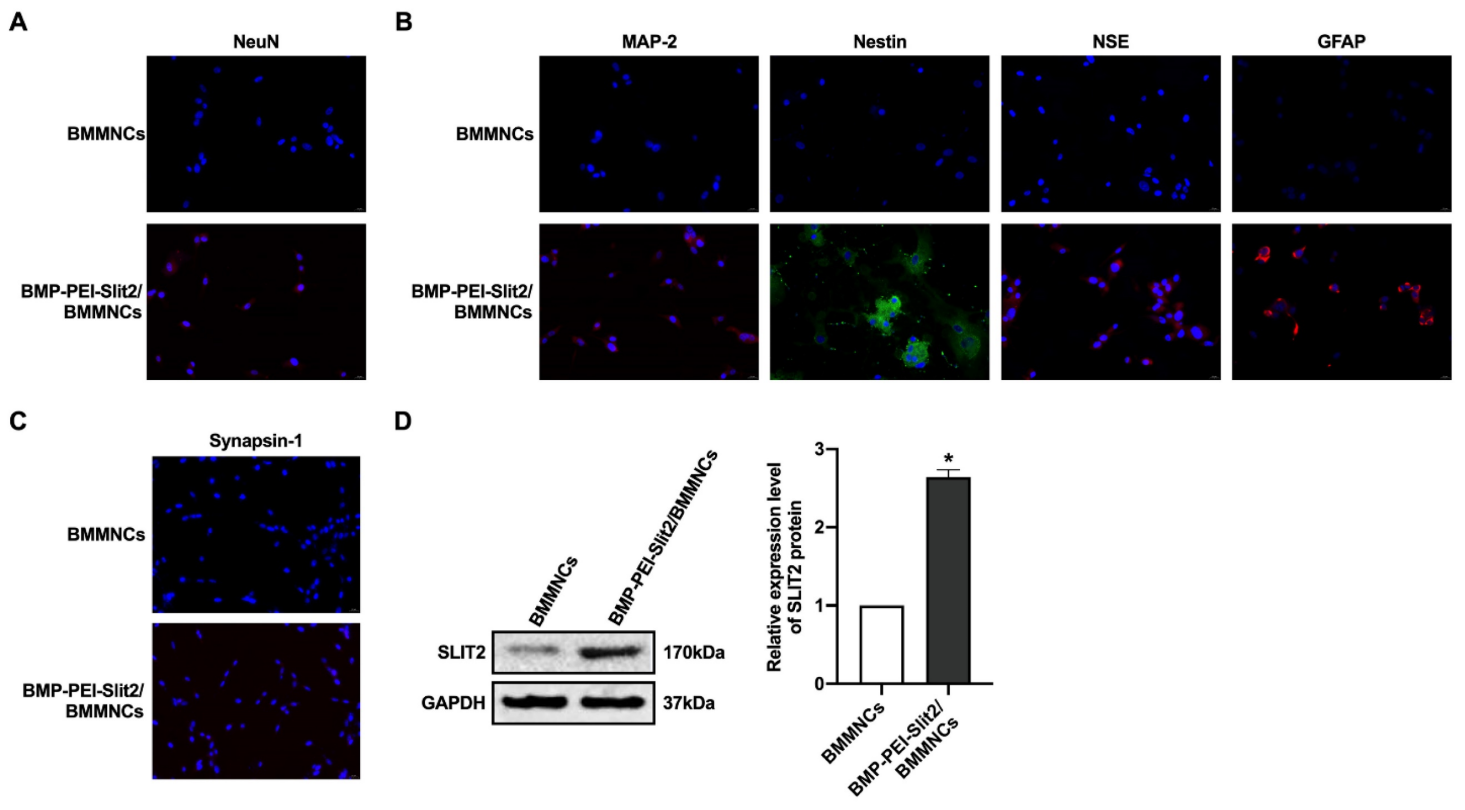
BMP-PEI-Slit2 induced BMMNC differentiation into neuron-like cells. The expression of NeuN after three days of in vitro culture of BMP-PEI-Slit2/BMMNCs (A), and the expression of MAP-2, Nestin, NSE, GFAP (B), and synapsin-1 (C) after 14 days of culture were detected by immunofluorescence. The expression of Slit2 in BMP-PEI-Slit2/BMMNCs cultured in vitro for 24 hours was detected by Western blot (D). *, *p* < 0.05 compare with BMMNCs group. BMMNC: Bone marrow mononuclear cell; BMP: superparamagnetic iron oxide nanoparticle; PEI: Polyethylenimine.

There was no significant change in BMMNC viability after BMP-PEI-Slit2 was co-cultured with BMMNCs for 24 hours (Figure 1A), and Slit2 mRNA expression significantly increased (Figure 1B). Under the inverted microscope, primary cells were small and round (Figure 1C); the cell bodies contracted after three days of induced culture, and neuronal synapse-like structures were observed around the cell bodies (Figure 1D); the cell bodies contracted significantly after seven days of culture, and typical unipolar or synapse-like structures of neurons were found around the cell bodies (Figure 1E); and the cell bodies shrunk and presented typical neural-like morphology after 14 days of culture with giant cell axons, which were connected into a network structure (Figure 1F). Meanwhile, BMP-PEI-Slit2 induced the expression of neuron-specific proteins in BMMNCs. BMMNCs expressed the neuronal nuclear protein NeuN after three days of *in vitro* culture of BMP-PEI-Slit2/BMMNCs (Figure 2A); and MAP-2, Nestin, NSE, GFAP (Figure 2B), and synapsin-1 (Figure 2C) were observed after 14 days of culture. Additionally, the Slit2 level significantly increased in BMP-PEI-Slit2/BMMNCs cultured *in vitro* for 24 hours (Figure 2D).

### BMP-PEI-Slit2/BMMNCs improved cognitive function in CCH rats

**Figure 3:**
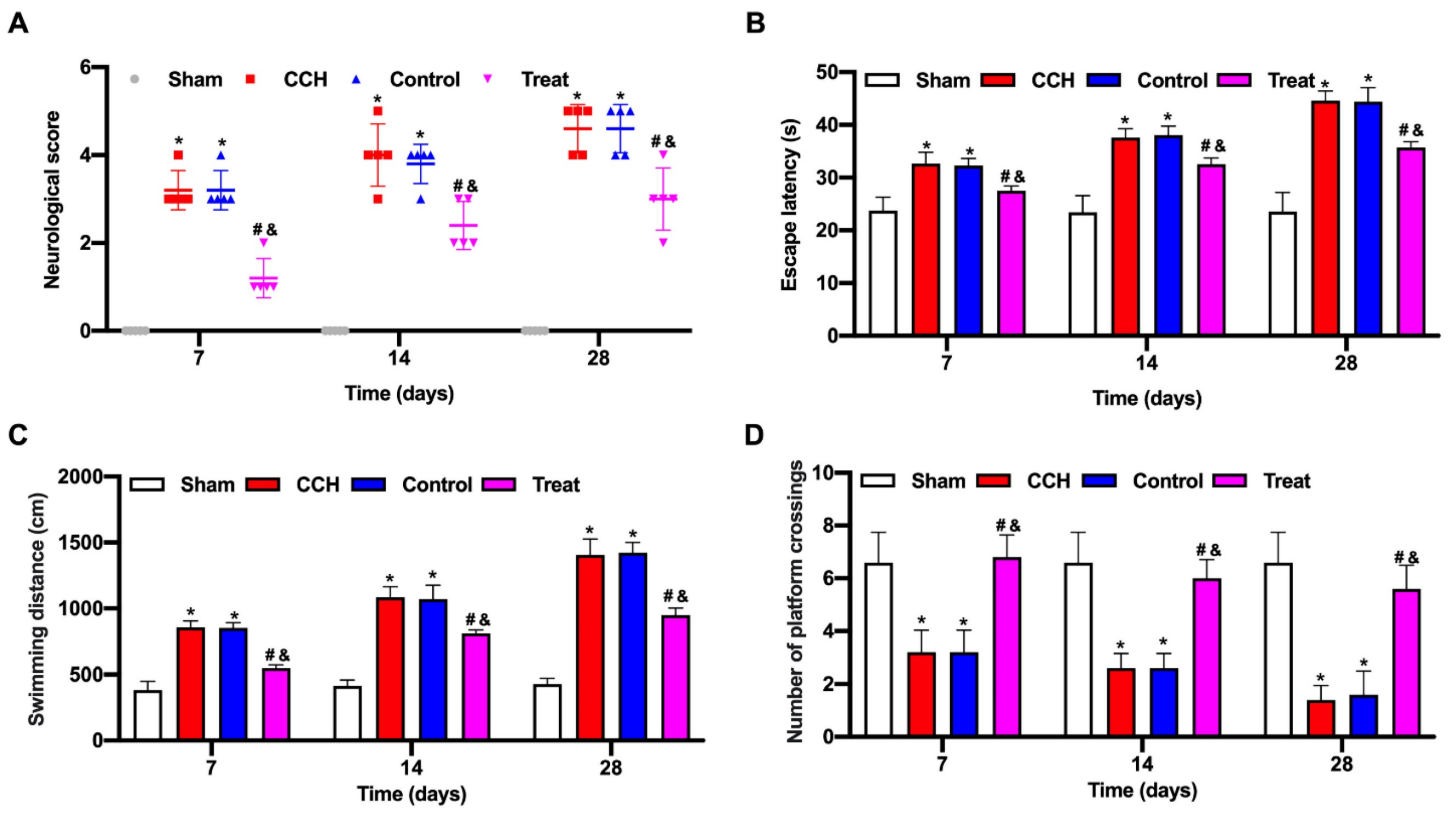
BMP-PEI-Slit2/BMMNCs improved cognitive function in CCH rats. A CCH rat model was established. BMP-PEI-Slit2/BMMNCs were injected into the tail vein and intervened with an external magnetic field. The cognitive function was observed at 7, 14, and 28 days, respectively. The neuronal score of rats was assessed using the Bederson method (A). The learning and memory abilities of rats were detected by directional navigation (B) and spatial exploration tests (C and D) in the Morris water maze. *, *p* < 0.05 compare with Sham group; #, *p* < 0.05 compare with CCH group; &, *p* < 0.05 compare with Control group. BMMNC: Bone marrow mononuclear cell; BMP: superparamagnetic iron oxide nanoparticle; CCH: chronic cerebral hypoperfusion; PEI: Polyethylenimine.

The neurological score of the CCH group significantly increased compared with that of the sham group, and the tail vein injection of BMP-PEI-Slit2/BMMNCs decreased the neurological score (Figure 3A). CCH rats showed prolonged escape latency and swimming distance and a reduced number of platform crossings, and the tail vein injection of BMP-PEI-Slit2/BMMNCs reversed the above effects (Figures 3C and 3D). Collectively, BMP-PEI-Slit2/BMMNCs relieved the cognitive dysfunction in CCH rats.

### BMP-PEI-Slit2/BMMNCs ameliorated brain tissue injury in CCH rats

**Figure 4:**
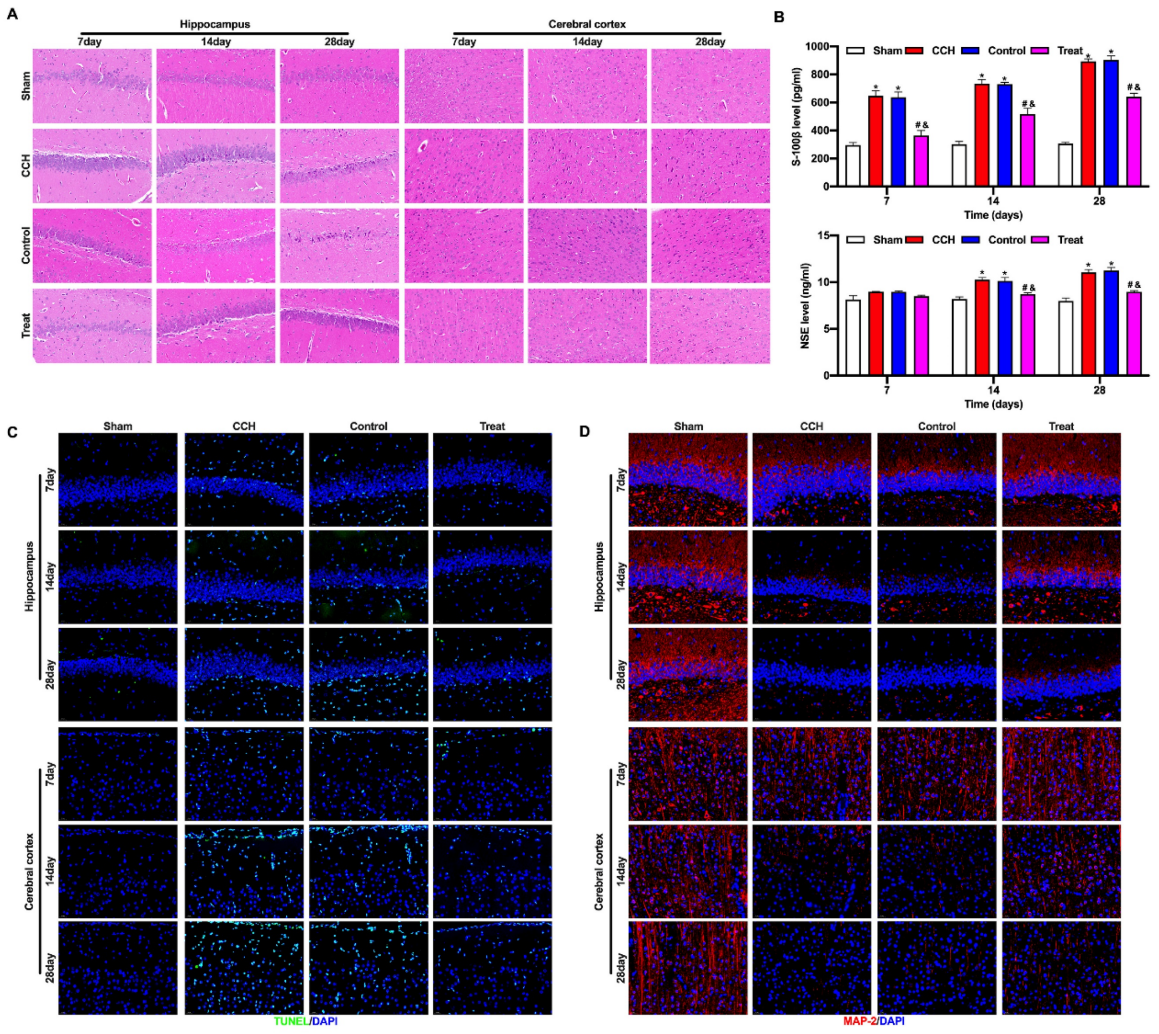
BMP-PEI-Slit2/BMMNCs ameliorated brain tissue injury in CCH rats. The pathological changes in the rat brain tissue were observed by HE staining (A). The expression levels of brain injury markers S-100β and NSE were measured by ELISA (B). The neuronal apoptosis in the frontal cortex and hippocampus of rats was observed by TUNEL assay (C). The expression of MAP2 in the frontal cortex and hippocampus of rats was detected by immunofluorescence (D). *, *p* < 0.05 compare with Sham group; #, *p* < 0.05 compare with CCH group; &, *p* < 0.05 compare with Control group. BMMNC: Bone marrow mononuclear cell; BMP: superparamagnetic iron oxide nanoparticle; CCH: chronic cerebral hypoperfusion; ELISA: enzyme-linked immunosorbent assay; HE: hematoxylin and eosin; PEI: Polyethylenimine; TUNEL: terminal dUTP nick-end labeling.

CCH rats had severe injury in the hippocampus and frontal cortex, pyknosis and degeneration of neurons in the frontal cortex, loose cell structure in the hippocampus, hippocampal atrophy, and neuronal degeneration and necrosis. Tail vein injection of BMP-PEI-Slit2/BMMNCs repaired the cell structure and neuronal morphology in the frontal cortex and hippocampus of CCH rats and prevented neuronal degeneration and necrosis (Figure 4A). The levels of brain injury markers S-100β and NSE w were significantly higher in the CCH group than in the sham group, and the tail vein injection of BMP-PEI-Slit2/BMMNCs decreased these levels (Figure 4B). Neuronal apoptosis in the frontal cortex and hippocampus of CCH rats significantly increased compared with that of the sham group, and the tail vein injection of BMP-PEI-Slit2/BMMNCs reversed this effect (Figure 4C). MAP2 expression in the frontal cortex and hippocampus of CCH rats was significantly lower than that in the sham rats, and the tail vein injection of BMP-PEI-Slit2/BMMNCs increased the expression (Figure 4D). These results indicated that BMP-PEI-Slit2/BMMNCs mitigated brain injury in CCH rats.

### BMP-PEI-Slit2/BMMNCs ameliorated brain injury in CCH rats by regulating the Slit2/Robo4 pathway

**Figure 5:**
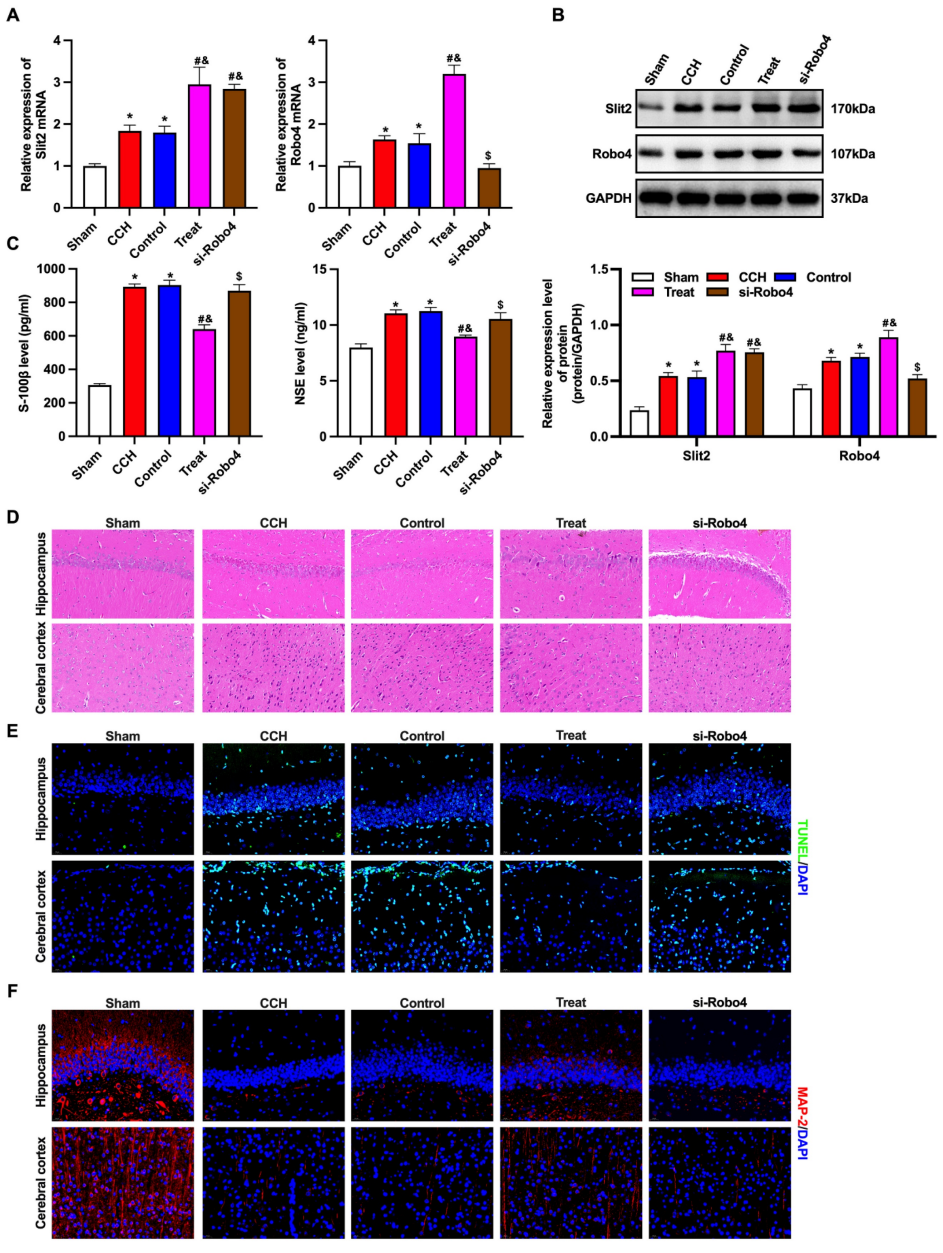
BMP-PEI-Slit2/BMMNCs ameliorated brain injury in CCH rats by regulating the Slit2/Robo4 pathway. The Slit/Robo pathway-related proteins Slit2 and Robo4 were detected by RT-qPCR (A) and Western blot (B). The expression of brain injury markers S-100β and NSE levels were measured by ELISA (C). The pathological changes in the rat brain tissue were observed by HE staining (D). The neuronal apoptosis in the frontal cortex and hippocampus of rats was observed by TUNEL assay (E). The expression of MAP2 in the frontal cortex and hippocampus of rats was detected by immunofluorescence (F). *, *p* < 0.05 compare with Sham group; #, *p* < 0.05 compare with CCH group; &, *p* < 0.05 compare with Control group; $, *p* < 0.05 compare with Treat group. BMMNC: Bone marrow mononuclear cell; BMP: superparamagnetic iron oxide nanoparticle; CCH: chronic cerebral hypoperfusion; ELISA: enzyme-linked immunosorbent assay; HE: hematoxylin and eosin; PCR: polymerase chain reaction; PEI: Polyethylenimine; TUNEL: terminal dUTP nick-end labeling.

The Slit/Robo pathway-related proteins Slit2 and Robo4 were detected by RT-qPCR and Western blotting (Figures 5A and 5B). The expression levels of Slit2 and Robo4 mRNAs and proteins in the brain tissue of CCH rats significantly increased, and the levels further dramatically increased after tail vein injection of BMP-PEI-Slit2/BMMNCs, while Robo4 siRNA reversed this effect. Furthermore, Robo4 siRNA reversed the up-regulation of S-100β and NSE levels, prevented brain injury, facilitated apoptosis, and decreased MAP2 levels in the frontal cortex and hippocampus of CCH rats injected with BMP-PEI-Slit2/BMMNCs (Figures 5C-F). In short, BMP-PEI-Slit2/BMMNCs alleviated brain injury in CCH rats by activating the Slit2/Robo4 signaling pathway.

### BMP-PEI-Slit2/BMMNCs improved cognitive function in CCH rats by regulating the Slit2/Robo4 pathway

**Figure 6:**
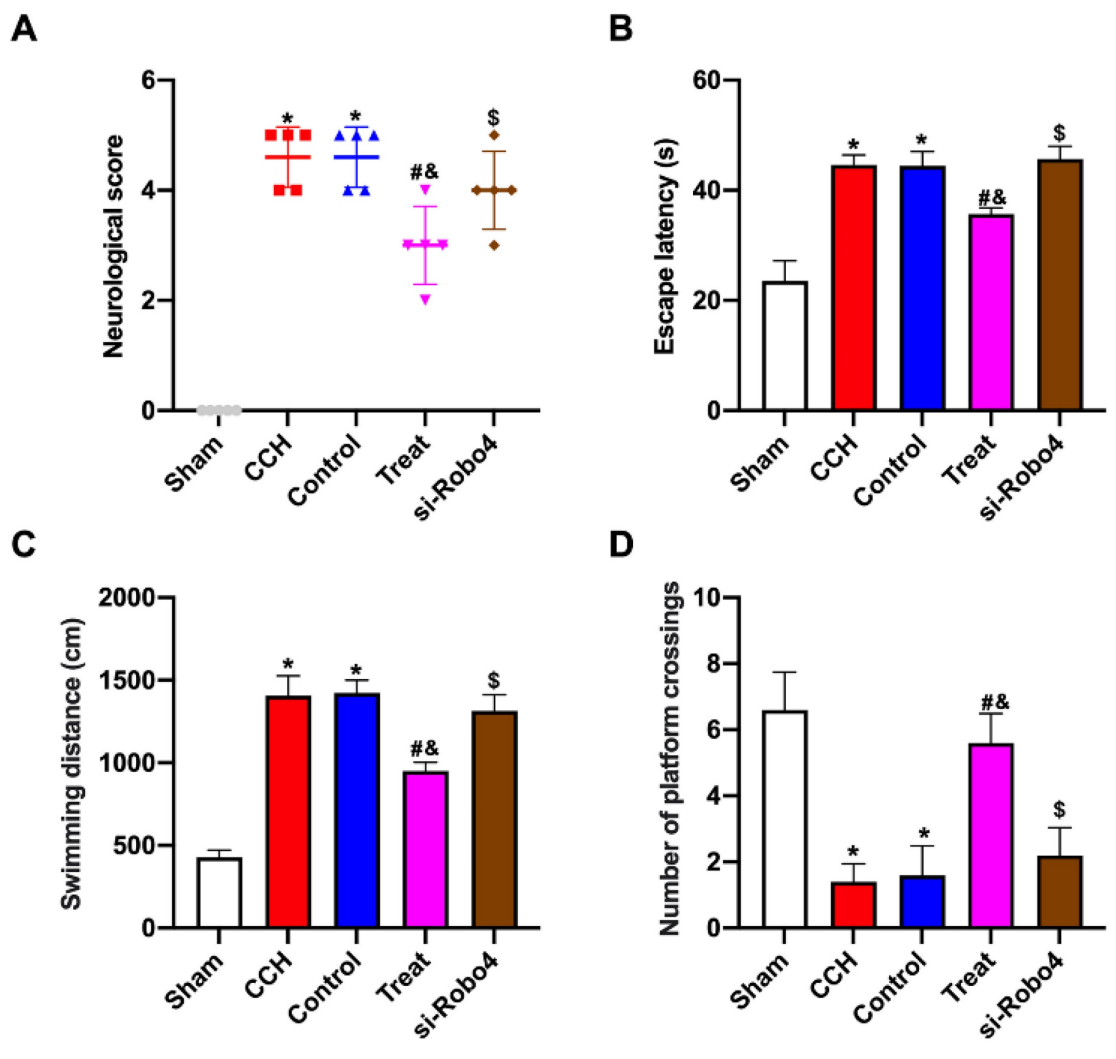
BMP-PEI-Slit2/BMMNCs improved cognitive function in CCH rats by regulating the Slit2/Robo4 pathway. The neuronal score of rats was assessed using the Bederson method (A). The learning and memory abilities of rats were detected by directional navigation (B) and spatial exploration tests (C and D) in the Morris water maze. *, *p* < 0.05 compare with Sham group; #, *p* < 0.05 compare with CCH group; &, *p* < 0.05 compare with Control group; $, *p* < 0.05 compare with Treat group. BMMNC: Bone marrow mononuclear cell; BMP: superparamagnetic iron oxide nanoparticle; CCH: chronic cerebral hypoperfusion; PEI: Polyethylenimine.

The neurological function scores of CCH rats treated with BMP-PEI-Slit2/BMMNC significantly increased after Robo4 siRNA administration (Figure 6A). The Morris water maze test showed that CCH rats treated with BMP-PEI-Slit2/BMMNCs exhibited significantly increased escape latency and swimming distance and decreased platform crossings after Robo4 siRNA administration (Figures 6B-D). These findings indicated that BMP-PEI-Slit2/BMMNCs relieved cognitive dysfunction in CCH rats by activating the Slit2/Robo4 signaling pathway.

## Discussion

This study first constructed a BMP-PEI-Slit2 complex based on the magnetotactic property, strong DNA adsorption ability of BMPs, high transfection efficiency, and difficult degradation of PEI to provide new ideas for studying a new potential treatment of CCH-induced cognitive impairment. By adding an external magnetic field intervention, we clarified that BMP-modified Slit2 migrated through BMMNCs to induce brain injury and cognitive dysfunction in CCH rats. Additionally, we explored the role of the BMP-PEI-Slit2/BMMNCs complex by blocking the Slit/Robo pathway.

Cognitive dysfunction caused by CCH is the primary cause of vascular dementia. Its mechanism and the pursuit of efficient intervention strategies have consistently been a research focus. BMMNCs are the general term for a cell population defined as the mononuclear cell component in the bone marrow. BMMNCs mainly consist of lymphocytes (such as B and T cells at different stages of maturation), precursor cells (like endothelial progenitor cells), and a small proportion of stem cells (such as hematopoietic stem cells, endothelial progenitor cells, and mesenchymal stem cells) 15. BMMNCs can complete their distribution in human body with blood circulation after intravenous transplantation by passing through the aorta and pulmonary circulation with blood flow. However, their role and mechanism in cerebral protection remain unclear. BMMNCs have relatively low efficacy on treating CCH due to their inadequate migration ratio to the ischemic brain after venous transplantation 15. The magnetic nanoparticles selected in our study had good application prospects in biomedical fields, and combining them with CCH-related genes effectively improved the efficiency of BMMNCs transplantation. Our results showed that BMP-PEI-Slit2/BMMNCs migration into the brain allevaited brain tissue injury and cognitive dysfunction and exerted neuroprotective effects by differentiating into mature neurons and decreasing apoptosis in brain tissue.

Slit is a classical axonal guidance molecule that was first discovered in *Drosophila*, and three species are well known in vertebrates, namely Slit1, Slit2, and Slit3, which are highly conserved in sequence 16. Slit1 is expressed exclusively in neural tissues 17. Slit2 is distributed in nervous tissue, kidney, lung, and endothelial cells 18. Slit3 is widely distributed in many tissues, including the brain and kidney, and is localized in mitochondria 19. Robo is a single transmembrane protein that acts as a receptor for the Slit with four isoforms in vertebrates, namely Robo1, Robo2, Robo3, and Robo4, of which Robo4 is specifically expressed in the vascular system 20. Jin et al. proposed that Slit2 and its receptors Robo1 and Robo4 mitigated edema and tissue damage during cerebral ischemia 13. Patel et al. found that Slit2 receptor was expressed on the surface of platelets, megakaryocytes, and their precursors and that Slit2 inhibited platelet aggregation and adhesion through experiments on patients and animal models 21. Therefore, Slit2 was a potent negative regulator of platelet function. It may reduce the inflammatory response in blood vessels, inhibit intimal hyperplasia and thrombosis, and block multiple injury events. Therefore, it worked as a target for preventing and treating diseases caused by thrombosis abnormalities, such as stroke and myocardial infarction. Park et al. used a rat model to demonstrate that Slit2 binding to its receptor attenuated cerebral ischemia/reperfusion injury by stabilizing the cytoskeleton and reducing neuronal death, including apoptosis and necrosis 22.

The expressions of endogenous Slit2 and its receptor Robo can be stimulated after cerebral ischemia/reperfusion, suggesting that the Slit/Robo pathway regulates endothelial cell migration, pathological angiogenesis, and vascular integrity during ischemic stroke 13,22. Taken together, Slit2 can prevent cerebral ischemia, reduce tissue injury, and promote tissue recovery after cerebral ischemia. Our findings showed that silencing Robo4 to block the Slit2/Robo signaling pathway reversed the ameliorative effects of BMP-PEI-Slit2/BMMNCs transplantation on brain tissue injury and cognitive dysfunction in CCH rats, indicating that BMP-PEI-Slit2/BMMNCs functioned by activating the Slit2/Robo signaling pathway.

Nevertheless, this study is limited. Only rats were tested. There may be obvious differences between humans and animals in terms of physiology, anatomy, and metabolism. Hence, the results should be further validated using human subjects.

## Conclusions

In summary, combining BMP with CCH-related gene Slit2 can effectively improve the efficiency of BMMNC transplantation in treatment. Additionally, this study may provide novel insights into stem cell applications, and have essential theoretical value and practical significance for understanding and preventing human diseases.

## Abbreviations

BMMNC: Bone marrow mononuclear cell
BMP: superparamagnetic iron oxide nanoparticle
CCH: chronic cerebral hypoperfusion
DMEM: Dulbecco's modified eagle medium
ELISA: enzyme-linked immunosorbent assay
HE: hematoxylin and eosin
PBS: phosphate-buffered saline
PCR: polymerase chain reaction
PEI: Polyethylenimine
TUNEL: terminal dUTP nick-end labeling
